# Therapeutic target database update 2016: enriched resource for bench to clinical drug target and targeted pathway information

**DOI:** 10.1093/nar/gkv1230

**Published:** 2015-11-17

**Authors:** Hong Yang, Chu Qin, Ying Hong Li, Lin Tao, Jin Zhou, Chun Yan Yu, Feng Xu, Zhe Chen, Feng Zhu, Yu Zong Chen

**Affiliations:** 1 Bioinformatics and Drug Design Group, Department of Pharmacy, and Center for Computational Science and Engineering, National University of Singapore, 117543, Singapore; 2 Innovative Drug Research Centre and College of Chemistry and Chemical Engineering, Chongqing University, Chongqing 401331, P. R. China; 3 College of Pharmacy, State Key Laboratory of Medicinal Chemical Biology and Tianjin Key Laboratory of Molecular Drug Research, Nankai University, Tianjin 300071, P. R. China; 4 Zhejiang Key Laboratory of Gastro-intestinal Pathophysiology, Zhejiang Hospital of Traditional Chinese Medicine, Zhejiang Chinese Medical University, No. 54 Youdian Road, Hangzhou 310006, China

## Abstract

Extensive drug discovery efforts have yielded many approved and candidate drugs targeting various targets in different biological pathways. Several freely accessible databases provide the drug, target and drug-targeted pathway information for facilitating drug discovery efforts, but there is an insufficient coverage of the clinical trial drugs and the drug-targeted pathways. Here, we describe an update of the Therapeutic Target Database (TTD) previously featured in NAR. The updated contents include: (i) significantly increased coverage of the clinical trial targets and drugs (1.6 and 2.3 times of the previous release, respectively), (ii) cross-links of most TTD target and drug entries to the corresponding pathway entries of KEGG, MetaCyc/BioCyc, NetPath, PANTHER pathway, Pathway Interaction Database (PID), PathWhiz, Reactome and WikiPathways, (iii) the convenient access of the multiple targets and drugs cross-linked to each of these pathway entries and (iv) the recently emerged approved and investigative drugs. This update makes TTD a more useful resource to complement other databases for facilitating the drug discovery efforts. TTD is accessible at http://bidd.nus.edu.sg/group/ttd/ttd.asp.

## INTRODUCTION

The modern drug development efforts (1–3) have led to the discovery and clinical testing of thousands of targeted agents, and the approval of a considerable number of drugs. These agents produce their therapeutic effects by modulating various targets in different biological and disease regulatory pathways. The knowledge of these agents, their efficacy targets and the targeted pathways is useful not only for the discovery and development of targeted therapeutics (4,5), but also for facilitating the research and development of systems pharmacology (6,7) aimed at the discovery of multitarget drugs (8) and drug combinations (9). Moreover, the relevant knowledge is useful for the further development and improvement of the tools used in the research and discovery of drugs, targets and system pharmacology.

While the comprehensive drug, target and drug-targeted pathway information is freely available in the established drug (10), efficacy target (11), pharmacology (12), bioactive compound (13), binding (14) and pathway (15) databases, there is an inadequate coverage of the number and the detailed information of the clinical trial drugs in these databases. As of August 2015, the number of explicitly labelled clinical trial drugs in these databases are 1130 (10), 3147 (11), 493 (12), 676 (13), 0 (14) and 0 (15), which is significantly less than that of current and discontinued clinical trial drugs (more than 9,528, as estimated in this study) searchable from the literature, public reports and clinical trial websites. The inadequate coverage of the clinical trial drugs in these databases can be further revealed by the analysis of their drug contents with respect to the reported drug clinical trial success rates. For instance, among these databases, the previous version of the Therapeutic Target Database (TTD)(11) contains the largest set of 3147 clinical trial drugs versus 2003 approved drugs. The ratio of the approved and clinical trial drugs is 63.6%, which is much larger than the reported 6.6–13.4% Phase I clinical trial to drug approval success rates (16,17), indicating the inadequate coverage of clinical trial drugs and targets in TTD.

There is also a lack of the coverage of the biological or disease regulatory pathways targeted by the clinical trial and investigative drugs. Moreover, while the drug-regulated pathway information may be obtained via the hyperlinks provided in some of these databases or by using the target gene ID to search the pathway databases, it is inconvenient to access the multiple drugs and targets involved in the regulation of the individual pathways. A substantial number of multitarget drugs produce their therapeutic effects via activities against multiple targets of the same pathway (18), and many drug combinations achieve synergistic therapeutic effects by modulating multiple targets in the same pathway (9). Therefore, a facility for the convenient access of the multiple targets and drugs involved in the regulation of individual pathways is highly useful for studying the mechanisms of multitarget and drug combination therapeutics.

To provide more comprehensive information about the clinical trial drugs, targets and pathways, and more convenient access of the targets and drugs associated with specific pathways, we made several major improvements to the TTD (http://bidd.nus.edu.sg/group/ttd/ttd.asp). The first is the significantly expanded coverage of the clinical trial targets and drugs to 723 targets and 9528 drugs. The second is the cross-linking of most of the TTD target and drug entries to the corresponding pathway entries of KEGG (15), MetaCyc/BioCyc (19), NetPath (20), PANTHER pathway (21), PathWhiz (22), Pathway Interaction Database (PID) (23), Reactome (24) and WikiPathways (25). Overall, 382 successful, 721 clinical trial and 330 research targets and 1144 approved, 4462 clinical trial, (including discontinued clinical trial drugs) and 14 065 investigative drugs (including preclinical drugs and drugs terminated at unspecified test stages) were cross-linked to their corresponding pathway entries. Each of these pathway entries typically contains the members of the pathway and some of the key regulators and downstream effectors (15,19–25). Therefore, the targets and drugs cross-linked to each pathway may play some roles in the regulation of the pathway or the activities of its effectors. The third is the convenient access of the multiple targets and drugs associated with each of the 302 KEGG, 211 MetaCyc/BioCyc, 26 NetPath, 134 PANTHER pathway, 99 PathWhiz, 222 PID, 578 Reactome and 524 WikiPathways pathway entries. Moreover, we added recently emerged, approved and investigative drug data to increase the TTD content to 397 successful and 1469 research targets, and 2071 approved and 17 803 investigative drugs, respectively. The statistics of our updated data is summarized in Table 1.

**Table 1.** Statistics of the targets, drugs and the cross-linked pathway entries in 2016 update of the TTD database

			2016 update	2014 update
Statistics of therapeutic targets	Number of all targets		2589	1959^a^
	Number of successful targets		397	388
	Number of clinical trial targets		723	461
	Number of research targets		1469	1110^a^
Statistics of drugs	Number of all drugs		31 614	20 667
	Number of approved drugs		2071	2003
	Number of drugs withdrawn from the market		154	0
	Number of clinical trial drugs		7291	3147
	Number of drugs discontinued in clinical trial		2237	498
	Number of preclinical drugs		357	163
	Number of drugs terminated in unspecified investigative stage		1701	0
	Number of investigative drugs		17 803	14 856
	Number of multitarget agents		26 368	20 818
	Number of drug combinations		115	115
Statistics of targets linked to their corresponding pathway entries	Number of successful targets linked to the number of pathway entries	KEGG	248–191	0 - 0
		Metacyc/Biocys	52–92	0 - 0
		Netpath	126–22	0 - 0
		PANTHER	148–89	0 - 0
		PathWiz	115–68	0 - 0
		PID	132–176	0 - 0
		Reactome	312–352	0 - 0
		WikiPathways	341–392	0 - 0
	Number of clinical trial targets linked to the number of pathway entries	KEGG	484–202	0 - 0
		Metacyc/Biocys	71–101	0 - 0
		Netpath	333–23	0 - 0
		PANTHER	312–96	0 - 0
		PathWiz	127–71	0 - 0
		PID	380–212	0 - 0
		Reactome	588–545	0 - 0
		WikiPathways	671–469	0 - 0
	Number of research targets linked to the number of pathway entries	KEGG	199–205	0 - 0
		Metacyc/Biocys	35–63	0 - 0
		Netpath	119–22	0 - 0
		PANTHER	145–97	0 - 0
		PathWiz	55–62	0 - 0
		PID	158–196	0 - 0
		Reactome	221–412	0 - 0
		WikiPathways	262–389	0 - 0
Statistics of drugs linked to their corresponding pathway entries	Number of approved drugs linked to the number of pathway entries	KEGG	863–191	0 - 0
		Metacyc/Biocys	148–97	0 - 0
		Netpath	409–22	0 - 0
		PANTHER	646–93	0 - 0
		PathWiz	463–69	0 - 0
		PID	454–178	0 - 0
		Reactome	974–354	0 - 0
		WikiPathways	1140–395	0 - 0
	Number of clinical trial drugs linked to the number of pathway entries	KEGG	3302–205	0 - 0
		Metacyc/Biocys	410–140	0 - 0
		Netpath	2125–25	0 - 0
		PANTHER	2347–105	0 - 0
		PathWiz	1210–86	0 - 0
		PID	2274–215	0 - 0
		Reactome	3901–570	0 - 0
		WikiPathways	4287–489	0 - 0
	Number of research drugs linked to the number of pathway entries	KEGG	9828–244	0 - 0
		Metacyc/Biocys	2853–149	0 - 0
		Netpath	6907–24	0 - 0
		PANTHER	8904–118	0 - 0
		PathWiz	5178–90	0 - 0
		PID	7259–213	0 - 0
		Reactome	12 788–560	0 - 0
		WikiPathways	14 491–500	0 - 0

^a^The number of research targets in the 2014 release is revised from that reported in the previous publication because of removal of redundant target entries.

## CLINICAL TRIAL DRUGS AND TARGETS

Clinical trial drugs represent special classes of therapeutic agents in advanced development stages, the knowledge of these drugs, their targets and the targeted pathways is very important for facilitating the future drug discovery efforts. Hence, there is a particular need to expand the coverage of the clinical trial drugs, targets and the targeted pathways. The clinical trial drugs and their efficacy targets were searched by the following procedure. First, we searched the PhRMA medicines in the 2009–2014 development reports and the 2014–2015 drug pipeline reports from the websites and annual reports of 183 companies. We then evaluated some of the clinical trial drugs in the earlier (2004–2010) versions of several commercial databases against the publicly accessible literature, company reports and company announcements to select those drugs reported in the public sources. The sources of the collected drugs are provided in Supplementary Table S1. For all the newly and previously collected drugs, we searched their synonyms or alternative names, removed the duplicates and further checked their clinical trial against the records in the ClinicalTrials.gov results database (26), company reports and company announcements. This procedure gave rise to 9528 drugs. These drugs were further divided into two groups, one without and the other with a report of the drug being discontinued in clinical trials in the publicly accessible literature or public announcements. The clinical phase of these drugs has been frequently provided in these multiple sources, which was recorded. For a drug with multiple clinical trial phase records, the highest phase was regarded as its clinical phase regardless of whether or not it has been discontinued in clinical trials.

The target of each drug was determined by the following procedure. For the drugs with a single target reported in these sources, each target was tentatively regarded as the efficacy target of the respective drug. For those drugs without target information or with more than one target (including different targets of the same drug reported in different sources), we conducted additional literature search for finding or verifying their efficacy targets linked to the clinically tested therapeutics using the established criterion of efficacy targets (27) that we have used in the development of the TTD database (28). An efficacy target is defined (27) as a protein, DNA, RNA or membrane component unambiguously involved in the initiation or progression of a disease, directly modulated by a therapeutic agent at sufficient level of potency that is typically <500 nM (ideally <100 nM) in biochemical assays (in some cases, drugs of μM potencies may show adequate potencies in cell-based, *in vivo* and clinical studies), and the claimed therapeutic activities have been further confirmed by additional biochemical assay and strong cell-based and *in vivo* studies linking the target to the drug action. For each target, its drug with the highest clinical phase was considered as the clinical phase of the target.

### THE DRUG REGULATED PATHWAYS AND THE CROSS-LINKING OF THE TTD ENTRIES TO THE ENTRIES OF THE PUBIC PATHWAY DATABASES

Drug action depends not only on the direct modulation of its target but also on other regulatory factors and the activities of the effectors of the complex biological networks and physiological systems (29). Advances in systems biology and bioinformatics have revealed some of these mechanisms at the network and physiological levels (9,30–33), which open more opportunities for the development of new polypharmacology strategies in the more effective treatment of the diseases (29,34). These coupled with the knowledge of drug-regulated pathways, including those of the clinical trial and investigative drugs as well as those of the approved drugs, are highly useful for facilitating the development of the novel polypharmacology-based drug discovery.

Moreover, a number of clinically used and tested drugs target multiple targets in the same pathway (e.g. Lapatinib, Afatinib, Dacomitinib and Neratinib inhibiting EGFR and HER2 in ErbB pathway, BEZ-235, XL-765, GDC-0980 and PF-05212384 inhibiting mTOR and PI3K in PI3K/AkT/mTOR pathway and CKI-27, RO-5126766, RG7304 and RG7388 inhibiting Raf and Mek in MAPK/ERK pathway)(18). One of the main mode of actions of the synergistic drug combinations is related to the modulation of multiple targets in the same pathway (9). Therefore, the information of the targets in and the drugs against the same pathway is highly useful for facilitating the discovery and the studies of the mechanisms of multitarget and drug combination therapeutics.

A number of targets can be found in the pathway entries of such public pathway databases as KEGG (15), MetaCyc/BioCyc (19), NetPath (20), PANTHER pathway (21), PathWhiz (22), PID (23), Reactome (24) and WikiPathways (25) databases. These targets may be the members, regulators or effectors of the biological networks in these pathway entries. Drug actions against some of these targets may have thus some effect on the activities of the respective pathways, and the cross-linking of the targets and drugs to their respective pathway entries may facilitate the discovery (29,34) and studies (9,30–33) of drugs, multitarget agents and drug combinations from the pathway regulation perspectives.

The cross-links of targets and drugs to the respective pathway entries were established by the following procedure. We firstly searched Uniprot database (35) to obtain the Uniprot ID of every target, from which each target and its drugs were directly linked via Uniprot cross-links to the corresponding KEGG (15) pathway entry or entries. We then mapped the Uniprot ID of every target to those of the members of the PANTHER pathway, PathWiz database, PID, Reactome and WikiPathways pathway entries. The cross-links of each target and its drugs to the corresponding members of the MetaCyc/BioCyc, NetPath and WikiPathways entries were established based on the match of the target's gene name with those in the gene name list of the respective pathway entries. Overall, we were able to link 381 successful targets and 1144 approved drugs, 721 targets and 4694 drugs in clinical trials, and 326 targets and 14 065 drugs in investigative stages to 302 KEGG, 211 MetaCyc/BioCyc, 26 NetPath, 134 PANTHER pathway, 99 PathWhiz, 222 PID, 578 Reactome and 524 WikiPathways pathway entries, respectively.

## ILLUSTRATIVE EXAMPLES OF THE SEARCH OF A TARGET, A DRUG AND THE CROSS-LINKED PATHWAY ENTRIES

A user can search for the information of a target in the ‘Search for targets’ field of the TTD homepage by inputting keywords such as a target name ‘Glucokinase’ and then clicking the ‘Search’ button on the right-hand side, which leads to the particular target page (Supplementary Figure S1), wherein, the user can find the information about the target type (in this case clinical trial target), synonyms, targeted diseases, biochemical class (in this case kinase), the function, drugs and the pathway entries of which the target is a member, regulator or effector. In the ‘Drug(s)’ section, a user can click the ‘Drug Info’ button behind each drug name to access the respective TTD drug page described below.

One may search for the information of a drug in the ‘Search for drugs’ field in the TTD homepage by inputting keywords such as a drug name ‘Buparlisib’, and then clicking the ‘Search’ button on the right-hand side, which leads to the particular drug page (Supplementary Figure S2), wherein the user can obtain the information about the drug structure (2D, 3D, InChI, INChIKey and SMILES), the drug-making company (in this case Novartis), the disease indication (disease and the corresponding international disease ICD9 and ICD10 codes), the drug status (in this case Phase III), the Pubchem compound ID, the Pubchem substance ID and its targets (in this case PI3K alpha, beta, gamma and delta). In the structure section, one can download the 2D or 3D structure of the drug by right-clicking the 2D-Mol or 3D-Mol link, respectively. In the ‘Target’ section, one can click the ‘Target Info’ button to access the respective TTD target page described above.

**Figure 1.**
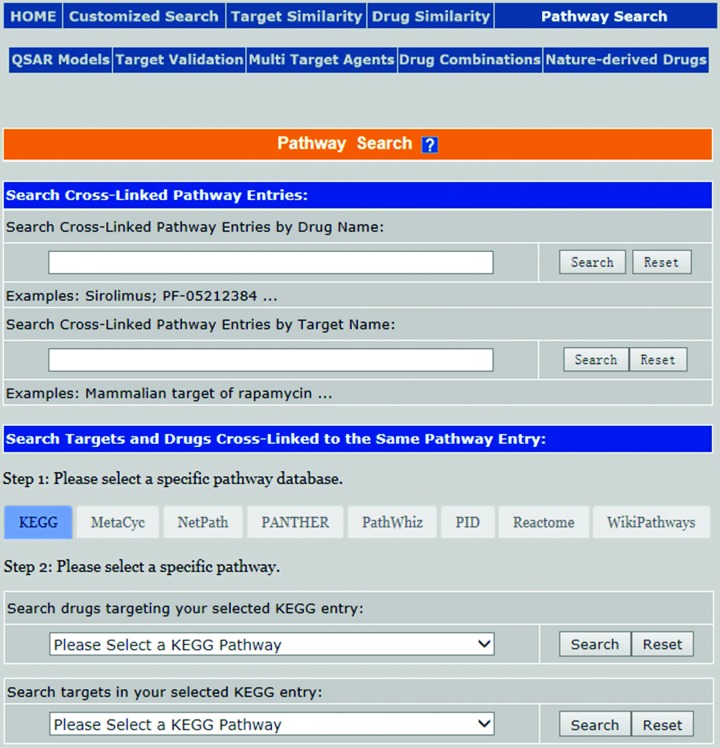
The pathway affiliation search page of the therapeutic target database.

A pathway search page was introduced for accessing the pathway entries cross-linked to a specific target or drug and for finding the targets or drugs cross-linked to the same user-selected pathway entry. This page (Figure 1) is accessible by clicking the ‘Pathway Search’ button in the upper right corner of the TTD homepage. The upper part of the page is the ‘Search Cross-Linked Pathway Entries’ field, wherein, the user can search the pathway entries cross-linked to individual targets and drugs by inputting a specific target name (e.g. Glucokinase) or drug name (e.g. Buparlisib) in the ‘Search Cross-Linked Pathway Entries by Target Name’ or ‘Search Cross-Linked Pathway Entries by Drug Name’ sub-field followed by the click of the ‘Search’ button in the respective search sub-field, which leads to the pathway entries cross-linked to the target (Supplementary Figure S3) or the drug (Supplementary Figure S4). The lower part is the ‘Search Targets and Drugs Cross-Linked to the Same Pathway Entry’ field, wherein, the user can search the targets and drugs cross-linked to the same pathway entries of a specific pathway database by clicking on the button of a particular pathway database (e.g. KEGG) followed by the selection of the particular pathway entry (e.g. Thyroid hormone synthesis) in the pathway entries manual, which leads to the page showing the list of targets (Supplementary Figure S5) or the drugs (Supplementary Figure S6) cross-linked to the same user-selected pathway entry.

## CONCLUDING REMARKS

The need for improved drug discovery productivity (36,37) has led to intensifying efforts in drug discovery by employing advanced technologies (1–3), novel therapeutic strategies such as polypharmacology (6,7), monoclonal antibodies (38) and RNA therapeutics (39), and the knowledge of the drug-like properties (40), target druggability features, and the systems-level profiles (9,30–33) learned from the studies of the approved and clinical trial drugs and targets. The enriched information and search facilities in TTD complements the other established drug (10) and pathway (15,19–25) databases for providing the comprehensive information about the drugs, targets and the targeted pathways in facilitating the research and drug discovery efforts.
